# ECMO-supported intervention for chronic centrally occluded pulmonary arteries

**DOI:** 10.1007/s12928-026-01308-9

**Published:** 2026-06-06

**Authors:** Hesham Salah El-Din Taha, Omar Younis, Mirna Mamdouh

**Affiliations:** 1 Department of Cardiovascular Medicine, Faculty of Medicine, Kasr Al-Ainy, Cairo University, Cairo, Egypt Al-Manial 11956; 2 Cardiology Department, National Heart Institute, Cairo, Egypt

**Keywords:** Chronic thromboembolic pulmonary hypertension, ECMO, Pulmonary Balloon Angioplasty

We report a 55-year-old West African male presenting with progressive exertional dyspnea and fatigue of 4-month duration. CT pulmonary angiography demonstrated a large saddle embolus with complete occlusion of the left pulmonary artery (LPA) and subtotal occlusion of the right pulmonary artery (RPA). Catheter-directed thrombolysis and mechanical thrombectomy were attempted with limited success, including failed LPA recanalization. The patient was subsequently diagnosed with chronic thromboembolic pulmonary hypertension (CTEPH) and discharged on apixaban 5 mg twice daily. This diagnosis and interventions were performed in February 2025 at a remote institution in patient’s home country.

The patient presented to our hospital later in June 2025 with cardiogenic shock and hypoxemia (blood pressure 85/55 mmHg, oxygen saturation 81%). Echocardiography demonstrated severely dilated right ventricle with profoundly reduced systolic function and extremely elevated right ventricular systolic pressure. Given these findings, cardiothoracic surgeons deemed pulmonary endarterectomy unsuitable at this stage. Although earlier surgical evaluation might have been considered, the rationale for prior management decisions remains unknown as care was at another remote institution.

The patient consented to catheter-based intervention. Two sessions were performed (Videos 1 & 2, Figs. 1 and 2) with veno-arterial ECMO support. By the second session, the pulmonary artery pressures decreased significantly from 121/73 mmHg (mPAP 89 mmHg) to 84/47 mmHg (mPAP 59 mmHg).

**Fig. 1:**
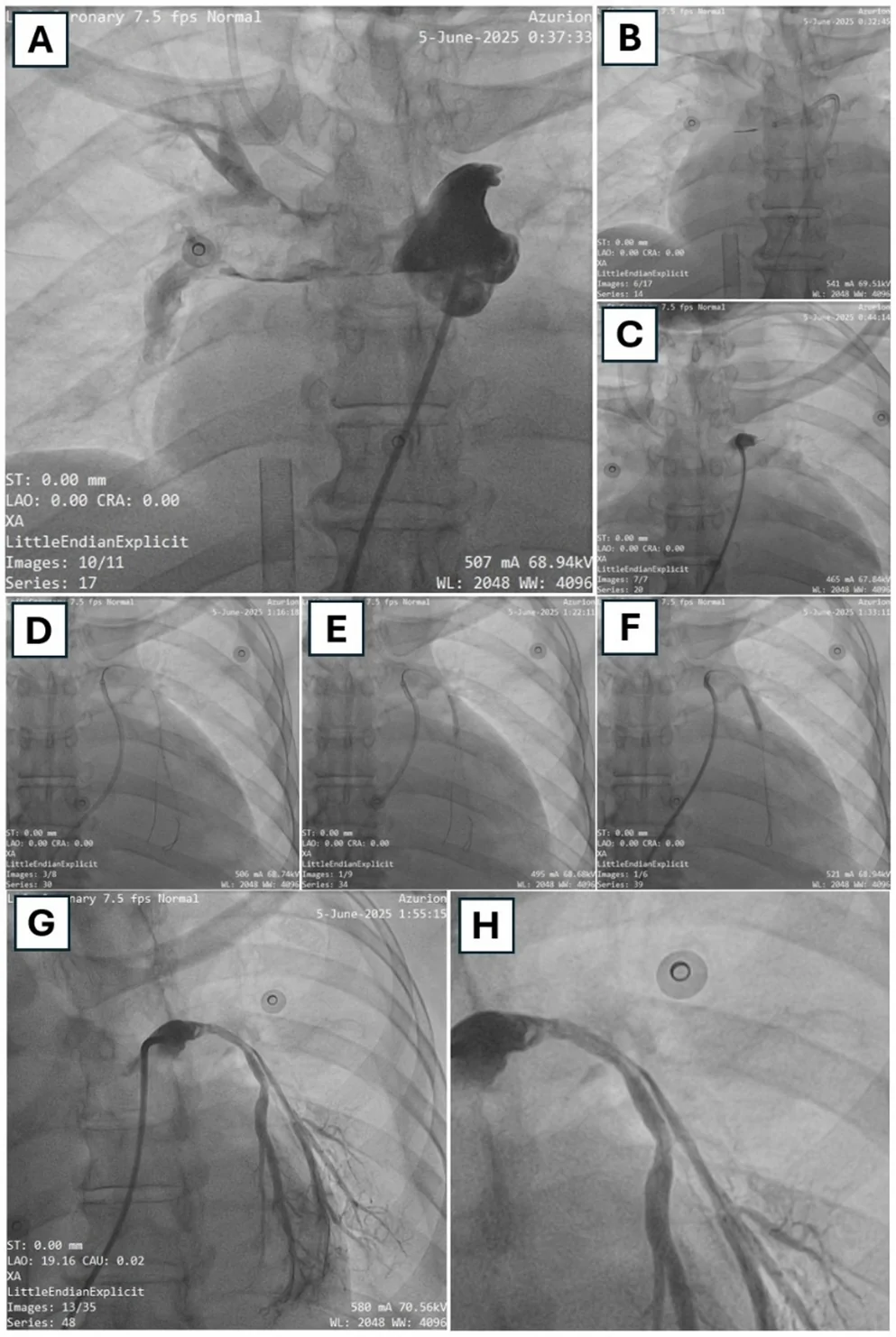
Session One: Stepwise endovascular recanalization of pulmonary arterial occlusion. **A** Pulmonary angiography demonstrating subtotal occlusion of RPA and its major branches, and total occlusion of LPA. **B** Mechanical aspiration of thrombus in RPA using the Penumbra Indigo^®^ CAT8 Aspiration System (Penumbra Inc., USA). **C** Aspiration attempt in LPA. **D & E** Successful traversal of occluded LPA segment using Pilot 50 and Pilot 200 coronary guidewires (Abbott, USA), followed by balloon angioplasty with Sprinter Legend™ balloons (1.5 × 20 mm and 2.5 × 20 mm). **F** Further lesion expansion using an NC Solarice™ balloon (4.0 × 20 mm; Medtronic, USA). **G & H** Final angiographic views demonstrating restored perfusion to lower branches of LPA

**Fig. 2:**
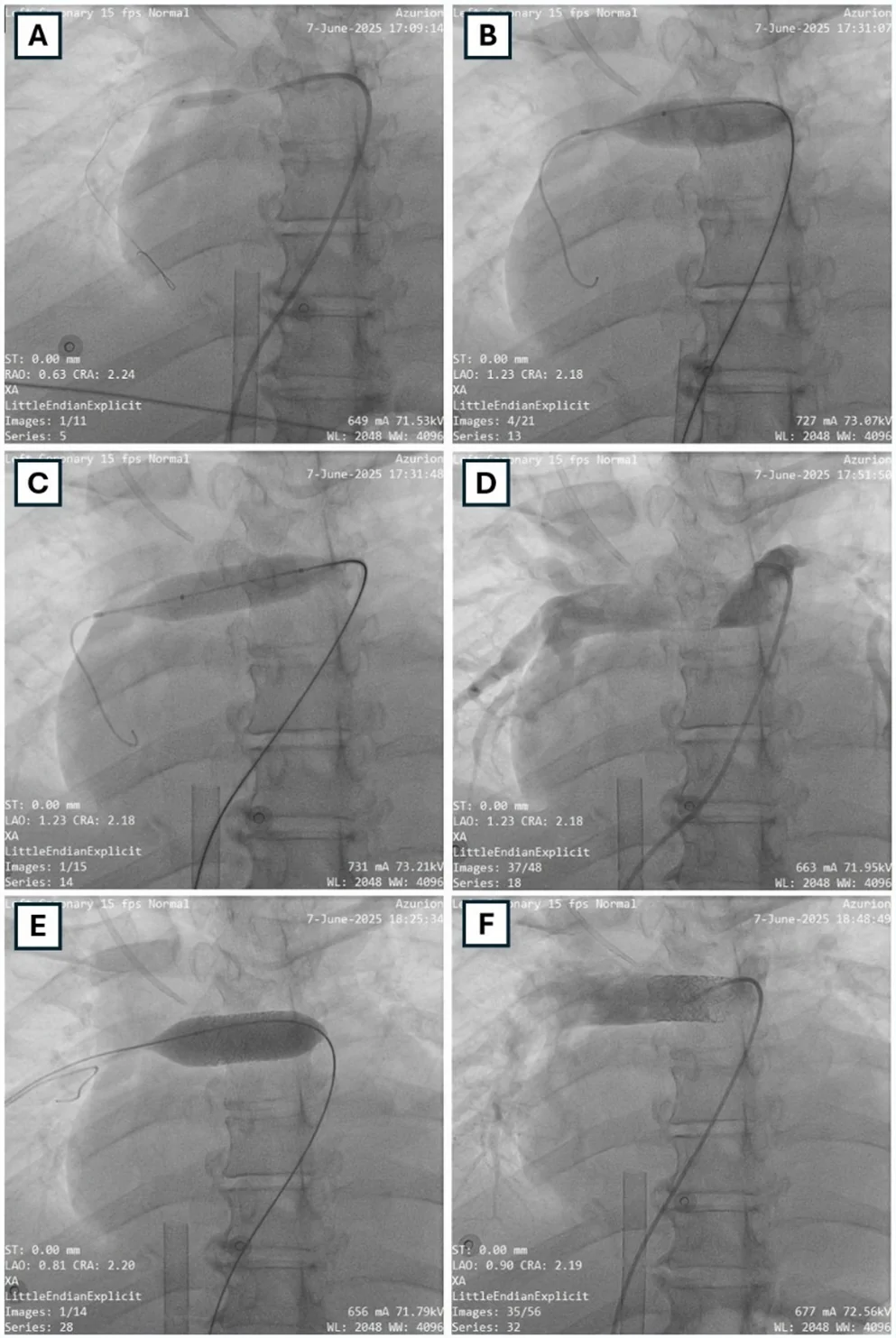
Session Two: Endovascular revascularization of a subtotally occluded RPA using balloon pulmonary angioplasty (BPA) and stent implantation. **A** Initial dilatation of the proximal segment with an NC Solarice™ balloon (5.0 × 15 mm). **B** Further expansion using an Atlas Gold™ balloon (14 × 40 mm; BD, USA) in proximal RPA. **C** Balloon angioplasty performed more distally within RPA. **D** Post-BPA angiogram showing improved contrast flow and restored perfusion in RPA and its distal branches. **E** Proximal stenting with BeGraft™ Aortic Stent (18 × 38 mm; Bentley, Germany) deployed over a Straight PTFE Amplatz ST 0.035/260 guidewire (Boston Scientific, USA). **F** Final angiographic views demonstrating optimal stent expansion, excellent strut apposition, and contrast flow across RPA and its major branches

The post-procedural course was complicated by reperfusion injury requiring mechanical ventilation, pneumonia, and vascular access bleeding. He was discharged on apixaban, riociguat, bosentan, torsemide, and spironolactone, with improved functional status at follow-up.

## Supplementary Information

Below is the link to the electronic supplementary material.

Supplementary Material 1

Supplementary Material 2

Supplementary Material 3

## Data Availability

Data available on request from the corresponding author.

